# Testicular Evaluation Using Shear Wave Elastography (SWE) in Patients with Varicocele

**DOI:** 10.3390/jimaging9090166

**Published:** 2023-08-22

**Authors:** Sandra Baleato-Gonzalez, Iria Osorio-Vazquez, Enrique Flores-Ríos, María Isolina Santiago-Pérez, Juan Pablo Laguna-Reyes, Roberto Garcia-Figueiras

**Affiliations:** 1 University Hospital Complex of Santiago de Compostela, 15706 Santiago de Compostela, Spain; iria.osorio.vazquez@sergas.es (I.O.-V.); enrique.flores.rios@sergas.es (E.F.-R.); roberto.garcia.figueiras@sergas.es (R.G.-F.); 2 Epidemiology Service, General Directorate of Public Health, 15704 Santiago de Compostela, Spain; soly.santiago.perez@sergas.es; 3 Department of Radiology, Faculty of Medicine, Universidad de Valparaíso, Valparaíso 2360102, Chile; juanpablo.laguna@uv.cl

**Keywords:** shear wave elastography, stiffness, testicle, ultrasound, varicocele

## Abstract

Purpose: To assess the possible influence of the presence of varicocele on the quantification of testicular stiffness. Methods: Ultrasound with shear wave elastography (SWE) was performed on 48 consecutive patients (96 testicles) referred following urology consultation for different reasons. A total of 94 testes were studied and distributed in three groups: testes with varicocele (group A, n = 19), contralateral normal testes (group B; n = 13) and control group (group C, n = 62). Age, testicular volume and testicular parenchymal tissue stiffness values of the three groups were compared using the Kruskal–Wallis test. Results: The mean age of the patients was 42.1 ± 11.1 years. The main reason for consultation was infertility (64.6%). The mean SWE value was 4 ± 0.4 kPa (kilopascal) in group A, 4 ± 0.5 kPa in group B and 4.2 ± 0.7 kPa in group C or control. The testicular volume was 15.8 ± 3.8 mL in group A, 16 ± 4.3 mL in group B and 16.4 ± 5.9 mL in group C. No statistically significant differences were found between the three groups in terms of age, testicular volume and tissue stiffness values. Conclusion: Tissue stiffness values were higher in our control group (healthy testicles) than in patients with varicocele.

## 1. Introduction

Ultrasound is the technique of choice to evaluate the testicular parenchyma due to its broad accessibility, low cost and high diagnostic sensitivity [1]. Nonetheless, its specificity is quite low [2,3]. Therefore, it would be extremely useful to have new diagnostic techniques such as elastography that may improve the assessment of testicular pathology. Essentially, elastography takes advantage of changed elastic properties of tissues in various pathologies to yield qualitative and quantitative information that can be used for diagnostic purposes. A correlation has been observed between elasticity and tissue condition: normal tissues are relatively more elastic than stiffer pathologic tissues.

Apart from the testis, ultrasound elastography has demonstrated promising clinical applications for evaluating thyroid, breast, liver, prostate, lymph node and musculoskeletal tissues. In the case of the thyroid, a recent meta-analysis [4] demonstrated that the combined sensitivity and specificity of elastography were much higher than those of conventional ultrasonography in the differentiation of benign and malignant thyroid nodules. In breast lesions, elastography may improve the characterization of breast masses and may provide additional information on predicting breast cancer prognosis and response to therapy [5]. Furthermore, ultrasound elastography techniques have also allowed a non-invasive method of estimating the degree of liver fibrosis in chronic liver disease [6]. In the prostate, elastography improves both prostate lesion characterization and prostate cancer detection and provides guidance for biopsy [7]. Elastography may also help to differentiate normal or reactive lymph nodes from malignant ones based on their stiffness and homogeneity [8]. Finally, elastography has shown a promising role in the characterization and determination of the severity and follow-up treatment of different pathologic conditions of the musculoskeletal system [9].

In the testis, elastography has been used in the assessment of different pathologic entities of the testis including undescended testis, testicular torsion and infarction, testicular microlithiasis, tumors, infertility and varicocele [10].

Qualitative techniques such as strain elastography were initially used for testicular evaluation. These techniques enabled information to be obtained on the strain–deformation ratio after application of an external tissue pressure [11]. In general terms, healthy tissues reveal low elasticity whilst pathological tissues present high elasticity and low compression capacity. These findings can be represented in a mode B superimposed color scale image. The sensitivity and specificity of real-time elastography (RTE) to differentiate benign from malignant lesions varies from 59% to 89% and from 25% to 38%, respectively [12]. However, the broad variability of the technique and difficulty attaining uniform compression in the testicular parenchyma has limited its clinical use.

Quantitative techniques, such as shear wave elastography (SWE), that evaluate the speed of propagation of waves in the tissue have enabled the quantification of testicular stiffness. This reveals, in real time, anatomical images associated with color scale maps and elasticity modules of the regions of interest.

This technique is simple to perform, non-invasive and highly reproducible, which enables measurement of both the speed of wave propagation (m/s) and tissue elasticity (kilopascals, kPa [11]. Although its use is extended to other anatomical areas, its application to scrotal pathology is recent and there is little literature in this regard [13,14,15,16,17].

The purpose of this work was to evaluate the possible impact of the presence of varicocele on the quantification of testicular stiffness.

## 2. Materials and Methods

A prospective study (2018–2019) was performed in our hospital using an ultrasound Aplio 500 (Toshiba Medical System, Japan) with linear probe PLT-1005BT 10–14 MHZ and 2DSWE two-dimensional shear wave elastography.

Patients were referred following urology consultation for different reasons: 31 patients with infertility (64.6%), 10 patients with pain and discomfort (20.8%) and 7 patients (18.85) with scrotal swelling (Table 1).

Patients were informed of the technique’s characteristics and consent for study was obtained. The exclusion criteria were refusal to participate and the presence of a single testicle. This study was approved by the hospital’s ethics committee.

A total of 48 patients (96 testicles) were recruited to undergo mode B ultrasound and evaluation with SWE. One patient had a testicular prosthesis, and one testicle was excluded from this study because of a testicular tumor.

Varicocele was defined as present when ultrasound revealed serpiginous tubules posterior to the testicle in correspondence with pampiniform plexus veins with a diameter greater than 2–3 mm.

In total, 94 testicles were evaluated and classified into three groups: testicles with varicocele (group A, n = 19), normal contralateral testicles of varicocele patients (group B, n = 13) and normal bilateral testicles (group C or control, n = 44). Based on previously published literature, it was deemed that the various ultrasound findings detected (4 epididymal cysts, 2 dilatation of the rete testis, 3 microlithiasis, 1 adenomatoid tumor, 1 hydrocele) did not alter testicular stiffness and were therefore included in the control group.

Mode B Ultrasound

Mode B ultrasound was performed on all patients. Volume was calculated for each testicle using the distances from the three testicular axes multiplied × 0.52.

Elastography technique

To perform elastography, any pressure effect was avoided. The transducer was placed on the testicles with slight contact. Abundant gel was used to remove the air between the surfaces (transductor–skin) and was kept fixed during acquisition.

The stiffness information was displayed on a dual-mode image using shear wave elastography mode, with one half of the screen displaying a color-coded stiffness box (blue to red color scales superimposed on the mode B image), and the other half of the screen displaying the reconstructed wave front of shear waves. Shear wave velocity values were displayed numerically in either kPa or m/s units and simultaneously. The stiffness scale was adapted to the values encountered in the stiffness box and was fixed at 30 kPa.

Quantitative analysis

To obtain quantitative data from SWE, a circular region of interest (ROI) of 5 mm diameter was placed in the testicular parenchyma, avoiding the areas with distortion in the color map and the testicular mediastinum identified as a hypercogenic band on mode B. We carefully did not add mechanical pressure during acquisition, because pressure can modify stiffness values.

**Figure 1:**
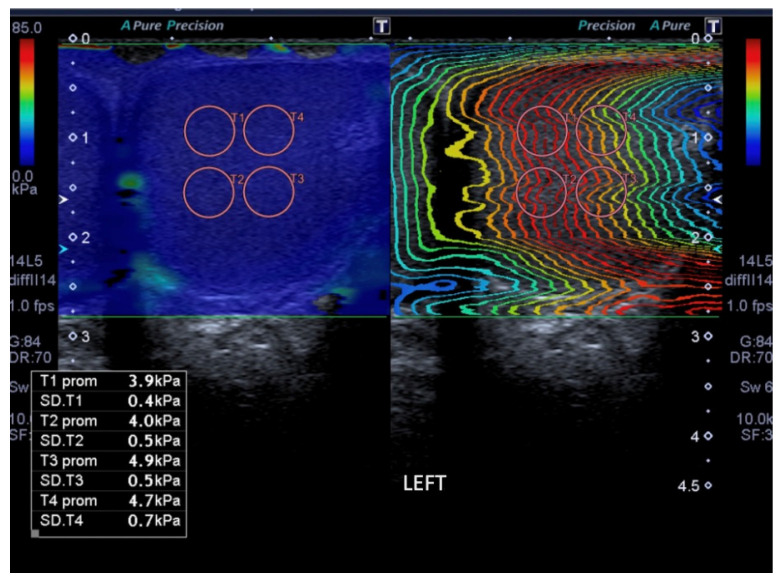
Elastography map of a normal testicle from a 40-year-old patient. Dual-mode image using shear wave elastography mode, with one half of the screen displaying the color-coded stiffness box (blue to red color scales superimposed on the mode B image), and the other half of the screen displaying the reconstructed wave front of shear waves. The ROIs are placed on the parallel waves.

Between three and five ROI measurements were taken for each testicle, in the center of the testicle in axial section (Figure 1). To record the stiffness value, a ROI was placed inside the color chart in correlation with the mode B image. Only the areas where waves were parallel were considered for statistical analysis.

The median of the three measurements was taken for statistical analysis.

A parameter was that interquartile range/median (IQR/M) was lower than 0.3 [17,18,19].

Statistical analysis

Qualitative variables were reported by frequency distribution, and quantitative variables by mean, standard deviation (SD) and quartiles (P25, P50 and P75). Age, testicular volume and SWE were compared among the three groups of testicles with the Kruskal–Wallis non-parametric test. A value of *p* < 0.05 was deemed statistically significant. Moreover, the correlation between age, testicular volume and SWE was analyzed graphically; the Pearson linear correlation coefficient was also calculated. Data were analyzed using Stata^®^ v14 statistical software.

## 3. Results

A total of 48 patients with a mean age of 42.1 ± 11.1 (range 26 to 74) were studied. Almost 80% were aged under 45. The main reason for consultation was infertility (Table 1).

Of the 48 patients, 16 (33%) had varicocele: 1 in the right testicle, 12 in the left testicle and 3 in both testicles. Therefore, 19 of the 94 testicles studied (20.2%) had varicocele (Table 2).

**Figure 2:**
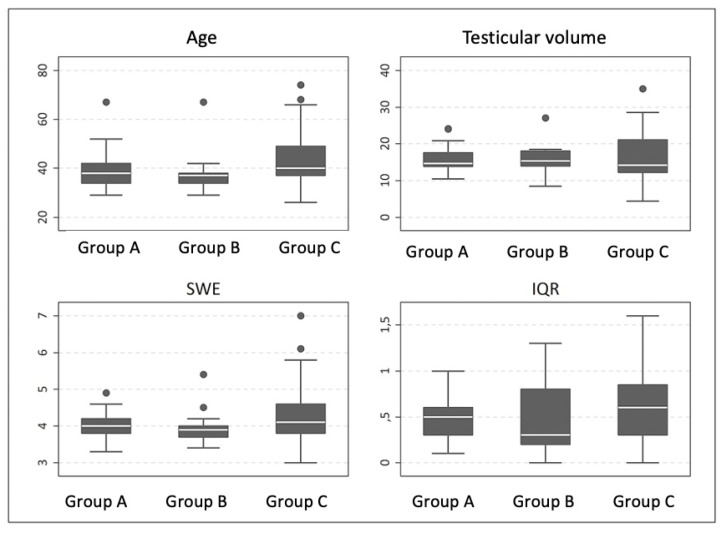
Age distribution (years), testicular volume (mL), SWE (kPa) and interquartile range (IQR) according to group, defined by the presence of varicocele.

Table 3 and Figure 2 show the age distribution, testicular volume and SWE according to the presence of varicocele.

No statistically significant differences were detected between the three patient groups regarding age (*p* = 0.09), testicular volume (*p* = 0.20) or tissue elasticity (*p* = 0.22).

**Figure 3:**
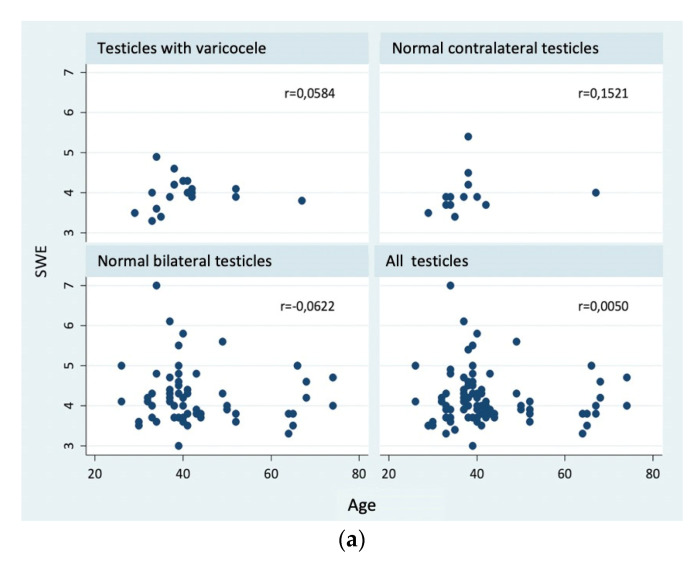

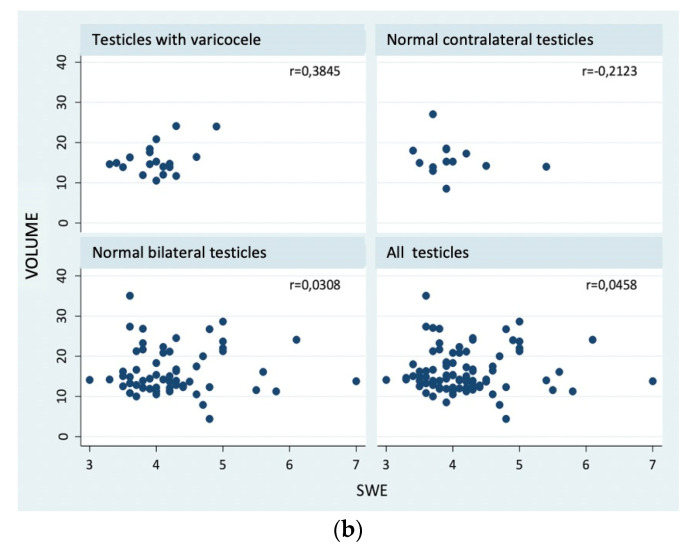
(**a**) Correlation graph of elasticity values (kPa) and age. (**b**) Correlation graph of volume (mL) and elasticity values kPa).

Nor was a significant correlation detected between age and SWE or volume and SWE, as shown in Figure 3a,b.

## 4. Discussion

Ultrasound is the imaging technique of choice to evaluate scrotal pathology, which includes the evaluation of male infertility [20,21]. Male infertility may occur for a broad range of reasons (hypogonadotropic hypogonadism, genetic abnormalities, testicular atrophy, tumors, etc.) but 40% to 50% of cases are idiopathic. Therefore, it is important to differentiate a benign from a malignant cause of infertility on imaging, as is the identification of the many causes of infertility that may be corrected, especially congenital abnormalities and disorders that obstruct sperm transport and varicocele [20,21,22,23,24].

Elastography, which includes both strain and SWE, provides complementary information on tissue properties compared to conventional ultrasonography [11]. SWE enables quantification of tissue elasticity and avoids the operator variability from manual compression that can occur with strain elastography [11]. SWE has most commonly been performed in the thyroid and breast to characterize focal lesions [4,5,6]. The use of SWE has been more limited in the testis. Trottman et al. were the first to publish data on tissue elasticity. They reported topographic differences in testicular stiffness in a sample of 66 healthy patients. Testicular stiffness values were higher at the poles of the testicle than in the center (1.15 m/s compared to 0.90 m/s, respectively) [25]. These authors suggested that this outcome might be related to the testicle’s anatomical structure. The testicle is segmented by multiple small septa which splits it into up to 400 lobules interrupted by the rete testis (network that anastomoses the tubules in the testicular hilum). These lobules are larger and longer in the middle of testicles [26,27]. However, the testicular hilum has a greater stiffness after anisotropy created by the convergence of vessels and septa that enter the parenchyma. This finding can account for elastography maps acquired in the axial plane being more reproducible and with less artefacts than in the longitudinal plane, as revealed by the Rocher group [28].

In general, the published literature has revealed a positive correlation between testicular stiffness and age. Trottman et al. published elasticity values in the upper pole of 1.10 m/s and 1.20 m/s for patients aged under and over 40, respectively [25]. D’Anastasi et al. reported similar results with the use of elastography based on acoustic radiation force impulse, ARFI [29]. These ultrasound findings could be explained by testicular changes related to age, such as reduction in size and in the number of all kinds of germinal cells (including Sertoli and Leydig cells). Histologically, there is a reduction in the volume occupied by the seminiferous tubules, whilst the volume of the testicular interstitium remains constant [27]. Testicular involution is characterized from the pathological point of view by a gradual regression in epithelial height and diameter of the seminiferous tubules, a continuous decrease in the absolute number of Sertoli cells (responsible for producing testosterone), and a reduction in the number of spermatogonia [26,27,30]. However, our study did not reveal significant changes in testicular stiffness with the age of patients.

Morphological and histological changes similar to age-related changes have also been reported after vascular lesions in the testicle [31,32]. Zhang et al. found that elastography values in mice were modified after testicular torsion showing increased stiffness [32].

Elastography can also provide information on testicular function. Yavuz et al. studied the value of elastography with the ARFI technique in the prediction of male infertility. They reported a negative correlation between mean media testicular speed and sperm count. A value of 1.465 m/s was set to enable the discrimination of normozoospermia from oligozoospermia with a sensitivity and specificity of 75% [33]. Rocher et al. also studied testicular stiffness both in healthy patients and those with different kinds of infertility. They found that testicular stiffness was similar in normal patients and those with obstructive azoospermia (mean 2.4 Kpa), whilst patients with oligoasthenoteratozoospermia presented lower stiffness values (approximately 2.1 Kpa) [28]. These findings might be related to testicular volume, given that they detected a positive correlation between testicular volume and stiffness in the same group of patients.

Varicocele is the main treatable cause of infertility. However, the relationship between varicocele and infertility is unclear and there are various theories in this regard [21,34,35]. Our study found a minimally decreased elasticity or stiffness in the varicocele group (4 +/− 0.4 Kpa) compared to the control group (4.2 +/− 0.7 Kpa). However, these differences were not statistically significant. Other groups have reported contradictory results. Erdogan et al. compared the elasticity of 58 patients with varicocele (n = 96 testes) and 52 control patients (n = 104 testes). They found that testicles with varicocele showed higher mean propagation speed and elasticity values than in normal patients (1.83 m/s and 12.61 kPa compared to 1.67 m/s and 10.17 kPa, respectively) [34]. Moreover, a recent paper published by Turna et al. also found higher stiffness values in varicocele patients (4.77 Kpa) compared to the control group (3.79 Kpa) [35].

Histological studies have revealed a reduction in the size of seminiferous tubules, peritubular fibrosis and atrophy of Leydig cells in varicocele patients [36]. In this setting, a possible explanation for the fact that varicocele can lead to abnormal testicular stiffness could be that varicocele causes venous stasis and, therefore, increased testicular temperature. This feature could lead to damage in the parenchyma with testicular atrophy and reduced testicular function. However, although our study found lower volume values in testicles with varicocele (15.8 mL) compared to normal testicles (16.4 mL), no statistically significant differences were detected. Published data are contradictory when setting volume as a parameter that might reflect testicular damage related to varicocele [37]. Currently, a spermiogram is used as an indirect method to evaluate testicular damage. Therefore, SWE could be a non-invasive alternative technique given, that testicular stiffness increases with fibrosis. This hypothesis has been proven experimentally in animal models and would also be in accordance with that observed in undescended testicles, which also show an increase in testicular stiffness [16,31,38,39].

Regarding the severity of varicocele and SWE values, a statistically significant relationship has not been detected in the published literature [35].

Finally, the potential value of SWE in the characterization of testicular pathology is starting to be studied. Roy et al. found that in normal testicles (n = 358) a mean stiffness of 4.55 Kpa, was very similar to the value of 4.2 Kpa detected in our study. These authors also studied different testicular processes and found an obvious increase in elasticity in testicular tumors, with a mean value of 21 Kpa. Other benign conditions including orchitis and focal areas of fibrosis presented values of 9.48 Kpa and 31.55 Kpa, respectively [14,28].

In summary, the use of elastography opens new possibilities in the evaluation of testicular pathology including infertility. Its use is recommended as a new diagnostic tool in the multiparametric ultrasound exam of the testicle [40].

## 5. Conclusions

In conclusion, our study using mode B ultrasound and SWE showed differences, although they were not statistically significant, between tissue stiffness and testicular volume in patients with varicocele compared to those without it.

## Figures and Tables

**Table 1:** Distribution of patients according to age group and reason for consultation.

	n	%
**Age group**		
25–34	12	25.0
35–44	26	54.2
≥45	10	20.8
**Reason for consultation**		
Infertility	31	64.6
Pain	8	16.7
Other reason	9	18.8

**Table 2:** Distribution by groups according to the presence of varicocele.

	Group	n	%
Testicles with varicocele	A	19	20.2
Normal contralateral testicles	B	13	13.8
Normal bilateral testicles	C	62	66.0

**Table 3:** Age, testicular volume, SWE and IQR as a function of the group, defined by the presence of varicocele.

	n	Mean ± SD	Range	P25	P50	P75
**Age**						
A	19	40.4 ± 8.7	29.0–67.0	34.0	38.0	42.0
B	13	38.3 ± 9.3	29.0–67.0	33.5	37.0	39.0
C	62	43.7 ± 11.9	26.0–74.0	37.0	40.0	49.0
**Testicular volume (mL)**						
A	19	15.8 ± 3.8	10.5–24.1	13.9	14.7	17.6
B	13	16.0 ± 4.3	8.5–27.0	14.0	15.3	18.2
C	62	16.4 ± 5.9	4.4–35.0	12.2	14.2	21.1
**SWE (kPa)**						
A	19	4.0 ± 0.4	3.3–4.9	3.8	4.0	4.2
B	13	4.0 ± 0.5	3.4–5.4	3.7	3.9	4.1
C	61	4.2 ± 0.7	3.0–7.0	3.8	4.1	4.6

## Data Availability

The data presented in this study are available on request from the corresponding author. The data are not publicly available due to patient privacy reasons.
